# Suberoylanilide Hydroxamic Acid Induces Hypersensitivity to Radiation Therapy in Acute Myelogenous Leukemia Cells Expressing Constitutively Active FLT3 Mutants

**DOI:** 10.1371/journal.pone.0084515

**Published:** 2013-12-19

**Authors:** Xufeng Chen, Eric H. Radany, Patty Wong, Shenglin Ma, Kan Wu, Bing Wang, Jeffrey Y. C. Wong

**Affiliations:** 1 Department of Radiation Oncology, City of Hope Cancer Center, Duarte, California, United States of America; 2 Department of Radiation Oncology, The First People′s Hospital of Hangzhou Medical Group, Hangzhou, Zhejiang, China; UT MD Anderson Cancer Center, United States of America

## Abstract

Histone deacetylase inhibitors (HDIs) have shown promise as candidate radiosensitizer for many types of cancers. However, the mechanisms of action are not well understood, and whether they could have clinical impact on radiotherapy for leukemia is unclear. In this study, we demonstrate that suberoylanilide hydroxamic acid (SAHA) can increase radiosensitivity of acute myeloid leukemia (AML) cells through posttranslational modification of Rad51 protein responses and selective inhibition of the homology-directed repair (HDR) pathway. Our data also showed that AML cells with mutant, constitutively active FMS-like tyrosine kinase-3 (FLT3) were more radiation sensitive, caused by compromised non-homologous end joining (NHEJ) repair. Furthermore, SAHA-induced radiosensitization were enhanced in AML cells with expression of these FLT3 mutants. The results of this study suggest that SAHA, a recently approved HDI in clinical trials, may act as a candidate component for novel conditioning regimens to improve efficacy for AML patients undergoing radiotherapy and chemotherapy.

## Introduction

Total body irradiation (TBI) continues to be important part of conditioning regimens for acute myelogenous leukemia (AML) patients undergoing hematopoietic cell transplantation (HCT). Several randomized trials have demonstrated superior outcomes using TBI compared to non-TBI containing regimens. Randomized phase II trials have also demonstrated reduced relapse rates in AML with just moderately higher TBI doses [1,2]. However, overall survival was unchanged due to an increase in toxicities and treatment related mortality rates. Novel, more targeted radiotherapy strategies are clearly needed to reduce associated side effects and to further safely dose escalate with the potential to improve outcomes. 

Recently advanced technology using image-guided intensity modulated radiotherapy, referred as total marrow irradiation (TMI), allows delivering highly conformal dose distributions to large complex target shapes such as the bone/bone marrow, while simultaneously reducing dose to critical normal organs [3,4]. Using this approach, radiation sensitizers take on greater importance in the transplant setting, since dose deposition can now be controlled and redistributed preferentially to marrow and away from normal organs, resulting in selective radiosensitization of marrow compared to normal tissues. The benefits of a radiosensitization strategy with even modest effects will be further amplified due to selective targeting of dose and radiosensitization to marrow and other user specified target structures.

Although the molecular basis of radiation response is complex and multifactorial, the predominant mechanism by which therapeutic irradiation (IR) kills most tumor cells is through clonogenic death. DNA double-stand breaks (DSBs) are regarded as the specific lesions that initiate this lethal response [5], and the repair of DSBs is then critical in determining radiosensitivity [6]. In mammalian cells, radiation-induced DSBs are repaired by a complex mechanism involving several principle pathways: non-homologous end joining (NHEJ), homology-directed repair (HDR) and single-strand annealing (SSA). Targeting these DNA damage repair machinery have potential impact in cancer radiotherapy [7-9]. 

We recently found that Suberoylanilide hydroxamic acid (SAHA) modulated IR-induced formation of RAD51 nuclear focus at DNA damage sites, resulting in suppressed homology-directed DNA damage repair and enhanced radiosensitivity in irradiated multiple myeloma cancer cells *in vitro* [10]. RAD51 is a recombinase protein essential in repairing DNA DSBs and stalled replication forks by homologous recombination (HR) [11]. Overexpression of RAD51 has been found in the majority of human tumor cells, and levels of RAD51 are positively correlated with the aggressiveness and increased invasiveness of cancers [12,13]. Overexpression of RAD51 also leads to resistance to DSB-inducing therapies. Thus, therapies targeting RAD51’s downregulation have been used to inhibit tumor growth and sensitize cancer cells to radio- and chemotherapies [14]. In this study, we show that SAHA also induce RAD51-dependent radiosensitization in AML cells. Interestingly, we found SAHA-induced radiosensitization were further enhanced in AML cells expressing constitutively activated FMS-like tyrosine kinase-3 (FLT3). Results from this study strongly suggest that SAHA may serve as a candidate component for novel conditioning regimens to improve efficacy for AML patients undergoing radiotherapy and chemotherapy. 

## Materials and Methods

### Reagents

SAHA was obtained from NCI/NIH (Rockville, MD). Anti-DNA-PKCs (4F10C5), anti-phospho-histone H2A.X (γ-H2A.X, ser-139), anti-phosphotyrosine 4G10, anti-ubiquitin antibodies, and anti-acetyl-histone H4 serum were purchased from Upstate (Charlottesville, VA). Anti-RAD51 (H-92), anti-KU86 (H-300), anti-KU70 (E-5), anti-Mre11 (H300), anti-RAD50 (G-2) and anti-actin (C-2) antibodies were from Santa Cruz Biotech. Inc. (Santa Cruz, CA). Pooled SiRNA oligos for RAD51 and control SiRNA-A were also from Santa Cruz Biotech. Inc.. Anti-FLT3 (8F2) antibody was from Cell signaling Technology (Danvers, MA). Anti-DNA PKCs (phospho T2609) antibody was from abcam (Cambridge, MA). Lestaurtinib (CEP-701) was obtained from LC Laboratories (Woburn, MA). Plasmid EJ5-GFP, pDsRed-Express2-N1 and pUC18 were kindly provided by Dr. Jeremy Stark and Dr. Binghui Shen (City of Hope Beckman Research Institute, Duarte, CA). Enzymes EcoR I and I-SceI were from New England Biolabs (Ipswich, MA).

### Cell culture

Human AML cell lines THP1, KG1A, TF1 and MV4-11 were from the American Type Culture Collection (Manassas, VA). THP 1 and TF1 cells were maintained in ATCC-formulated RPMI-1640 medium (Manassas, VA) with 10% non heat-inactivated fetal bovine serum (Omega scientific, Tarzana, CA); 2ng/ml Human GM-CSF (Genzyme Corporation, Cambridge, MA) was added into RPMI-1640 for TF1 cells. KG1A and MV4-11 were maintained in IMDM from ATCC, with 20% non heat-inactivated fetal bovine serum. Human sarcoma U2OS cells (HTB-96, ATCC, Manassas, VA) integrated with the DR-GFP, SA-GFP, EJ5-GFP, and EJ2-GFP reporters [10,15] were maintained in DMEM medium with 2 mM L-Glutamine, 4.5 g/L glucose, and 10% non heat-inactivated fetal bovine serum (Omega scientific). 

### Engineering expression of FLT3 in TF1 cells

pcDNA3.1 construct encoding FLT3 with internal tandem duplications (ITD) in juxtamembrane domain was provided by Jonathan D. Licht (Northwestern University, Chicago, IL). Wild-type FLT3 was obtained from THP1 cells by RT-PCR, and cloned into pcDNA3.1 vector. Plasmids encoding point mutations of FLT3 (D835Y and D835V) were generated by mutagenesis PCR. All plasmid constructs were confirmed by sequencing. 

TF1 cells were transfected with different forms of FLT3, and pcDNA3.1 empty vector as control. Stable transfectants were selected by G418 in methylcellulose medium (STEMCELL Technologies Inc.). 

### Irradiation

AML cells were irradiated using a Mark I Cs-137 Irradiator (J.L. Shepherd Association, San Fernando, CA) at a dose rate between 1.20 to 1.26 Gy/min. Administered doses were validated using nanodot optically stimulated luminescence dosimeters (Landauer, Inc. Glenwood, Illinois).

### Clonogenic assay

AML cells in log-phase were suspended in 0.2 mL complete medium containing different doses of SAHA or DMSO as control, mixed with 2 mL methylcellulose medium, and plated at two densities in triplicate for clonogenic assays [10]. After 14 days of incubation, colonies consisting >50 cells were directly scored using an inverted microscope. Average numbers for survival colonies were plotted versus doses of SAHA to determine lethal doses (LD) for each cell line. 

To evaluate radiosensitivity, cells were seeded at 2×10^5^/mL for 24 hours, and treated with SAHA at the indicated concentrations, or DMSO as control; IR was delivered 16 hours later. 250 to 10000 cells were then plated in methylcellulose medium three hours later for clonogenic assays. Colony formation for each condition was calculated in relation to values obtained for untreated control cells. Mean inactivation doses were determined and the sensitizer enhancement ratio (SER) for HDAC inhibitor treatment was calculated as the ratio of mean inactivation dose_control_/mean inactivation dose_SAHA-treated_ [10]. 

In SiRNA experiments, THP1 cells were transiently transfected with RAD51 SiRNA or control SiRNA-A. After 24 hours, cells were pretreated with SAHA for 16 hours, and irradiated with indicated doses. Cells were then plated in methylcellulose medium three hours later after IR for clonogenic assay. 

### Immunofluorescence analysis

AML cells were collected by centrifugation, and washed once with Ca^++^/Mg^++^-free PBS. Cells were then fixed in 4% paraformadehyde. Immunofluorescence analyses were performed as previously reported [10]. Images were acquired with LSM 510 confocal microscope (Zeiss) with 40X objective and processed by Photoshop (Adobe). At least 100 cells from each experiment were selected at random and were counted to calculate the percentage of cells as “positive” for both target proteins and γ-H2A.X if they displayed >5 discrete dots in nuclei. Cells containing discrete merged dots per nuclei in cells that were scored as RAD51 or phosphor-DNA-PKCs foci-positive were counted as positive for co-foci. 

### DNA damage repair assay

1×10^5^ U2OS reporter cells were plated onto a 12-well plate and transfected the next day with 0.8 µg pCBASce [10], and 0.4 µg plasmid constructs encoding different types of FLT3, or pcDNA3.1 empty vector as control. Three hours later after the initiation of the transfection, cells were washed once with growth medium. When SAHA or CEP701 were applied, 200 nM SAHA or 50nM CEP701 were added, and DMSO was include as control. GFP positive cells were quantified 72 hours later by flow cytometric analysis Up to 5x10^4^ cells were counted for each sample.

### *In vivo* NHEJ assay

*in vivo* NHEJ assay was based on the reactivation of linearized plasmid as previously reported [16] with modification. Briefly, 1x10^6^ cells were co-transfected with 0.5 μg pUC18 substrate (linearized with EcoR I) and 0.1 μg pDsRed2-N1 (as transfection control) by using electroporation (Gene Pulse Xcell, Bio-Rad, Hercules, CA). Three hours after transfection, cells were irradiated and maintained in complete medium for 72 h. Plasmid DNA was recovered from the cells using miniprep kit (Qiagen) and used to transform competent bacterial DH5α cells and plated in LB-agar plates with ampicillin for pUC18 and kanamycin for pDsReD-Express2-N1. Bacterial colonies for re-circularized pUC18 were used to assess several repair parameters: NHEJ repair efficiency was determined by the total bacterial colonies produced for pUC18 versus colonies for pDsReD-Express2-N1; correct repair and misrepair of DSBs were represented by blue and white bacterial colonies, respectively; the misrepair frequency was estimated by the number of white colonies as a percentage of total colonies. For statistically analysis, recovered plasmid DNA from each experimental sample was used to perform at least 3 bacterial transformations in parallel.

As an alternative approach, cells were co-transfected with plasmid EJ5-GFP (linearized with I-SceI) and pDsReD-Express2-N1. After IR, cells were harvested 72 hours later and analyzed by Flow Cytometry (Fortessa Flow Cytometer, Fluofarma, Princeton, NJ) [17]. The ratio of GFP-positive cells to DsRed-positive cells was used as a measure of NHEJ efficiency. 

### Immunoblot assay

For immunoblot assay, whole cell lysates were prepared in RIPA buffer with mild sonication. 1 mM TSA and 5 mM nicotinamide were added into RIPA buffer for assays of acetyl-histone H4. For immunoprecipitation, 500 μg of cell lysate was incubated with 2 μg antibody at 4°C overnight in Catch and Release® V2.0 system (Millipore Corporation, MA).

### Median Effect Analysis

Median effect analysis was employed to quantify the interaction of SAHA and IR. For combined treatments, the ratio of IR dose to SAHA dose was kept constant based on the LD_50_, and cells were treated with increasing total doses. A plot of the log of the total dose versus log of the reciprocal of the fraction of cells affected minus 1 yielded linear plots. The slope and y-intercept from these plots were used to calculate the CI as described previously [18].

### Statistical Analyses

Statistical analyses were performed using the Student`s t-test. A p value <0.05 was considered as significant (*).

## Results

### SAHA induces radiosensitization in AML cells *in vitro*

**Figure 1:**
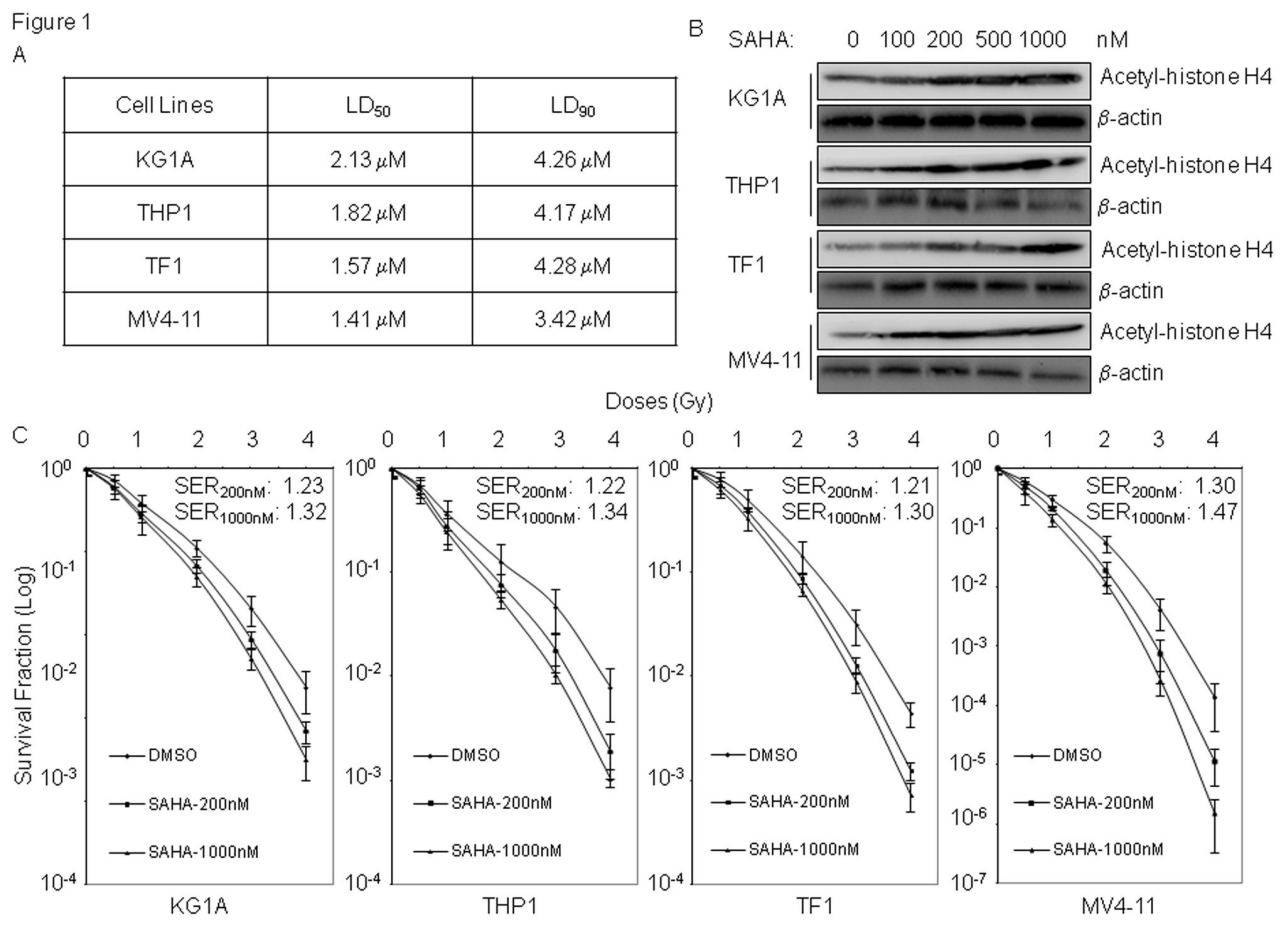
SAHA induces radiosensitization in AML cells. (A) LD_50_/LD_90_ values of SAHA on AML cells. AML cells were exposed to various concentrations of SAHA, and plated in methylcellulose medium for colony formation. LD_50_/LD_90_ values were determined based on the survival colonies. Data represent the average results from three independent experiments. (B) SAHA induces histone H4 acetylation. AML cells were exposed to SAHA for 16 hours. Whole cell extracts were prepared and analyzed by immunoblot assay for acetylation of histone H4. Anti-actin antibody was included for loading control. (C) SAHA induces radiosensitization in AML cells. AML cells in log phase were pretreated with 200nM or 1 μM of SAHA for 16 hours, and then irradiated at indicated doses. Clonogenic survival assays were performed in methylcellulose medium as described in materials and methods. Data represents the average of three experiments. Error bars indicate standard deviation.

We first determined the median lethal dose (LD_50_) and the effective doses of SAHA on histone acetylation in AML cells. Our results showed that the LD_50_'s of SAHA on tested AML cells ranged from 1.41 μM to 2.13 μM. Previous study has shown that blood cells of the monocytic lineage with expression of human carbonxylesterase-1 (hCE-1^+ve^) were more sensitive to SAHA [19], we also noticed that LD_50_ values of SAHA in hCE-1^+ve^ myelomonocytic tumor cell lines (THP-1 and MV4-11) were lower than that in hCE-1^-ve^ KG1A cell line. When cells were exposed to a serial of concentrations of SAHA, a minimum of 200 nM SAHA markedly increased acetylation of histone H4 in all cell lines (Figure 1A and B). 

We next tested the radiosensitization effects of SAHA on these AML cell lines. We found that pretreatment with both 200 nM and 1 μM of SAHA resulted in remarkable radiosensitization in all AML cell lines tested (Figure 1C). However, while exposure to 1 μM of SAHA significantly decreased plating efficiencies by 30-40% in these AML cell lines, treatment with 200 nM SAHA only decreased plating efficiency by 17% in MV4-11 cells, and no obvious cytotoxicities with reduced plating efficiencies were observed in KG1A, THP1 and TF1 cell lines (Figure S1). Thus, these results suggest that SAHA with minimal cytotoxic doses can sensitize AML cells to radiation treatment. 

### SAHA inhibits RAD51-mediated DNA damage repair in irradiated AML cells

**Figure 2:**
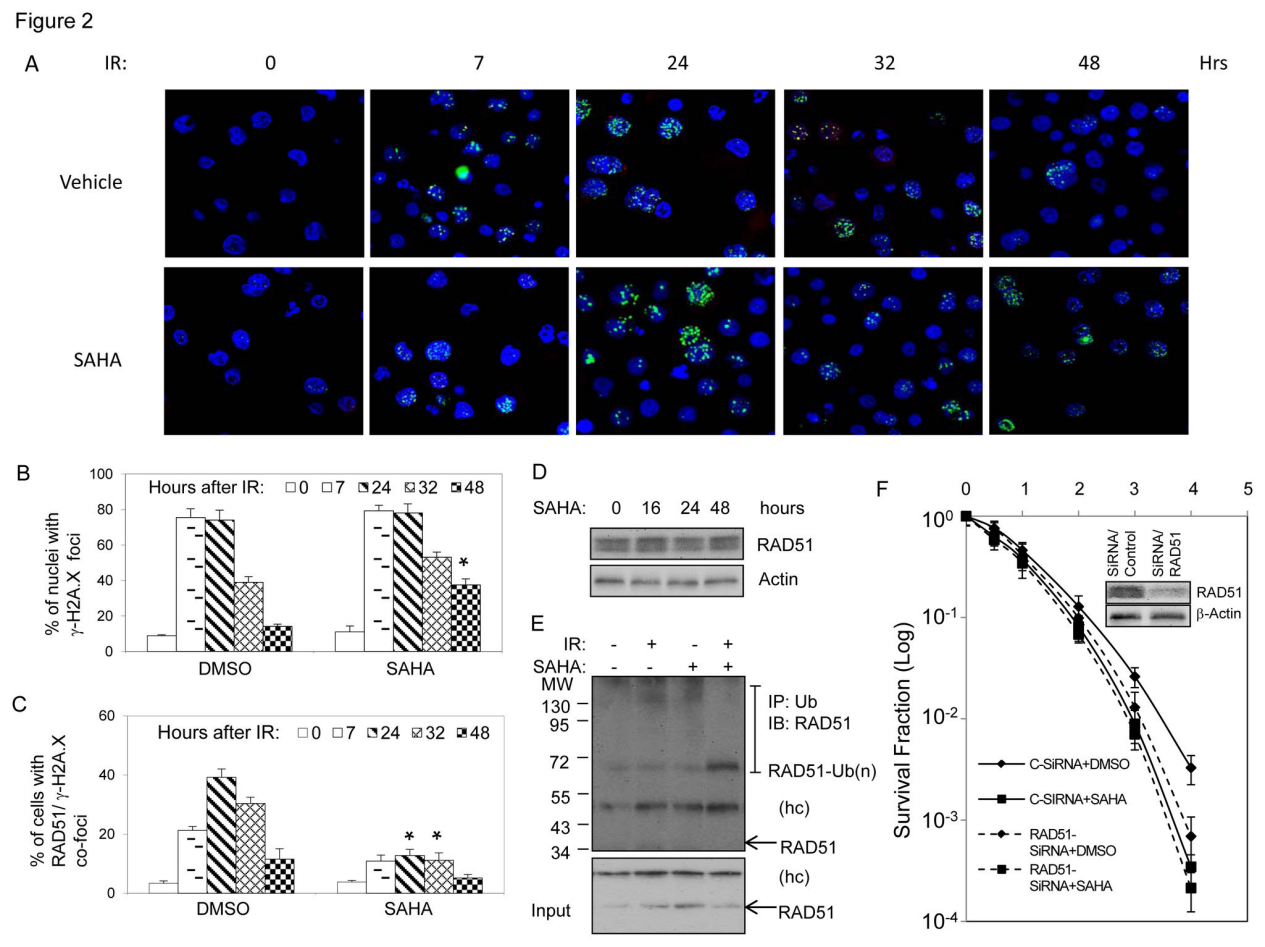
SAHA inhibits RAD51-medicated DNA damage repair in irradiated AML cells. (A) Representative images of nuclear RAD51 /γ-H2A.X co-foci in irradiated THP1 cells. THP1 cells were exposed to 200 nM SAHA for 16 hours and irradiated (1.2 Gy). Cells were then collected for immunofluorescence staining with anti-γ-H2A.X foci and anti-RAD51. (B and C) Diagram shows changes in the fraction of cells with γ-H2A.X foci (A) and RAD51/γ-H2A.X co-foci (B). (D) Effect of SAHA on expression of RAD51 protein. THP1 cells were exposed to 200 nM SAHA. Whole cell extracts were prepared and analyzed by immunoblot assay for RAD51 expression. Anti-actin antibody was included as a loading control. (E) Exposure to SAHA enhances RAD51 degradation in irradiated THP1 cells. After treatment with combination of SAHA (200nM) and IR (1.2 Gy), total cell lysates were collected from THP1 cells three hours post-IR, and analyzed for RAD51 degradation. Anti-ubiquitin antibody was used for immunoprecipitation, and anti-RAD51 antibody was used for immunoblot. (F) RAD51 mediates SAHA-induced radiosensitization. THP1 cells were transiently transfected with RAD51 SiRNA, or control-SiRNA-A. 24 hours later, cells were treated with combination of SAHA and IR, and were then plated in methylcellulose medium for clonogenic assay. Immunoblot shows the inhibitory effect of SiRNA transfection on RAD51 proteins in THP1 cells (samples collected 72 hours after transfection). Data represents the average of three experiments. Error bars indicate standard deviation. * indicates significance (P<0.05).

We previously reported that exposure to SAHA induced the persistence of γ-H2AX nuclear foci in irradiated multiple myeloma cells, which was associated with the inhibition of RAD51-dependent HDR [10]. To test whether SAHA exposure also affect IR-induced DNA damage repair in AML cells, we examined the kinetic changes of γ-H2A.X foci in irradiated AML cells. In THP1 cells, the cell fraction with γ-H2A.X foci dramatically increased, reaching 75% of cell population 7 to 24 hours after 1.2 Gy IR, and then decreased slowly over time. By 48 hours, the percentage of cells with residual γ-H2A.X foci dropped to 14.12 ± 1.25. Exposure to 200 nM SAHA for 16 hours slight increased the baseline percentage of γ-H2A.X foci positive cells from 8.8% to 11%, but had no obvious effects on the changes of γ-H2A.X foci formation within 24 hours in response to IR. However, the percentage of cells with residual γ-H2A.X foci after 48 hours in irradiated cells remained at a higher percentage (36.92 ± 4.09, p=0.013), indicating that SAHA exposure reduces repair capability for DNA DSBs and causes the persistence of lethal DNA damage in irradiated THP1 cells [20,21] (Figure 2A and B, and Figure S2A and B). 

We also examined the effects of SAHA on the formation of RAD51/γ-H2A.X co-foci in THP1 cells exposed to IR. In control cells, only a small fraction of THP1 cells (about 3%) showed subnuclear RAD51 foci (≥ five foci per nucleus), and no RAD51/γ-H2A.X co-foci were detected. After IR, the percentage for cells with nuclear RAD51/γ-H2A.X co-foci increased, achieving maximum level (39.55 ± 2.90) at 24 hours. As expected, IR-induced the formation of RAD51 foci were mostly apparent in γ-H2A.X foci positive cells, with < 5% of cells demonstrating RAD51 foci in the absence of γ-H2A.X foci. Exposure to SAHA significantly reduced nuclear formation of RAD51/γ-H2A.X co-foci in irradiated cells. For example, SAHA exposure resulted in 63.43% (p=0.0007) and 69.55% (p=0.0005) reduction in the percentage of cells with RAD51/γ-H2A.X co-foci at 24 hours and 32 hours after IR, respectively (Figure 2A and C, and Figure S2A and B). 

Similar effects of SAHA on the persistence of γ-H2A.X foci and the reduced formation of RAD51/γ-H2A.X were also observed in irradiated KG1A, TF1 and MV4-11 cells (Figure S2C and D). Notably, we found that MV4-11 cell had more baseline γ-H2A.X foci (15.45±0.80) when compared to other tested AML cells (7.58±1.94 for KG-1A and 8.88±0.96 for TF1, respectively), which might be caused by elevated level of reactive oxygen species (ROS) due to mutant FLT3/ITD in MV4-11 cells [22].

 We next tested the regulatory effects of SAHA on expression and modifications of RAD51 protein in THP1 cells. In these experiments, we also examined the effects of SAHA on the expressions of several other DSBs repair-related proteins. Our immunoblot assays showed that exposure to 200 nM of SAHA for up to 48 hours did not change the protein level of RAD51, and protein levels of DNA-pKcs, Mre11, Ku86, RAD50 and Ku70 neither. However, when cells were exposed to 1 µM SAHA for 16 hours, slight decreases were observed with protein levels of RAD51, Mre11, RAD50 and Ku70 (Figure S2E). We previously found that exposure to low dose of SAHA remarkably blocked IR-induced increase of RAD51 protein level, and treatment with MG132, a 26S proteasome inhibitor, restored SAHA-repressed RAD51 protein level and the formation of RAD51/γ-H2A.X foci in irradiated MM cells [10]. We thus hypothesize that SAHA may modulate RAD51 degradation in response to IR, which in turn results in the reduction of RAD51/γ-H2A.X co-foci formation in responses to IR-induced DNA damage [23,24]. Indeed, our results further showed that exposure to 200 nM of SAHA significantly increased the level of ubiquitin conjugated-RAD51 upon IR treatment (Figure 2E).

To verify the role of RAD51 on SAHA-induced radiosensitization in AML cells, we further determined the changes of radiosensitivity in THP1 cells with silencing of RAD51 protein. As shown in Figure 2F, blockage of RAD51 expression with targeting SiRNA increased the radiosensitivity of THP1 cells. When compared to control cells transfected with control SiRNA, the radiosensitization effect of SAHA was reduced in RAD51-silenced THP1 cells with SER value for 1.09 ± 0.02 (vs 1.23 ± 0.06 in control cells, p=0.026).

Taken together, these data suggest that SAHA exposure also inhibits DNA damage repair which is dependent on RAD51 response in irradiated AML cells.

### AML cells with constitutively activated FLT3 are more radiosensitive

Mutation in FLT3 receptor, in the form of both ITD and point mutations in the kinase domain, result in constitutive activation of FLT3 [25-27]; Studies demonstrated that activated FLT3 through acquisition of the FLT3/ITD led to a compromised NHEJ pathway and increased genomic instability [16,28]. 

**Figure 3:**
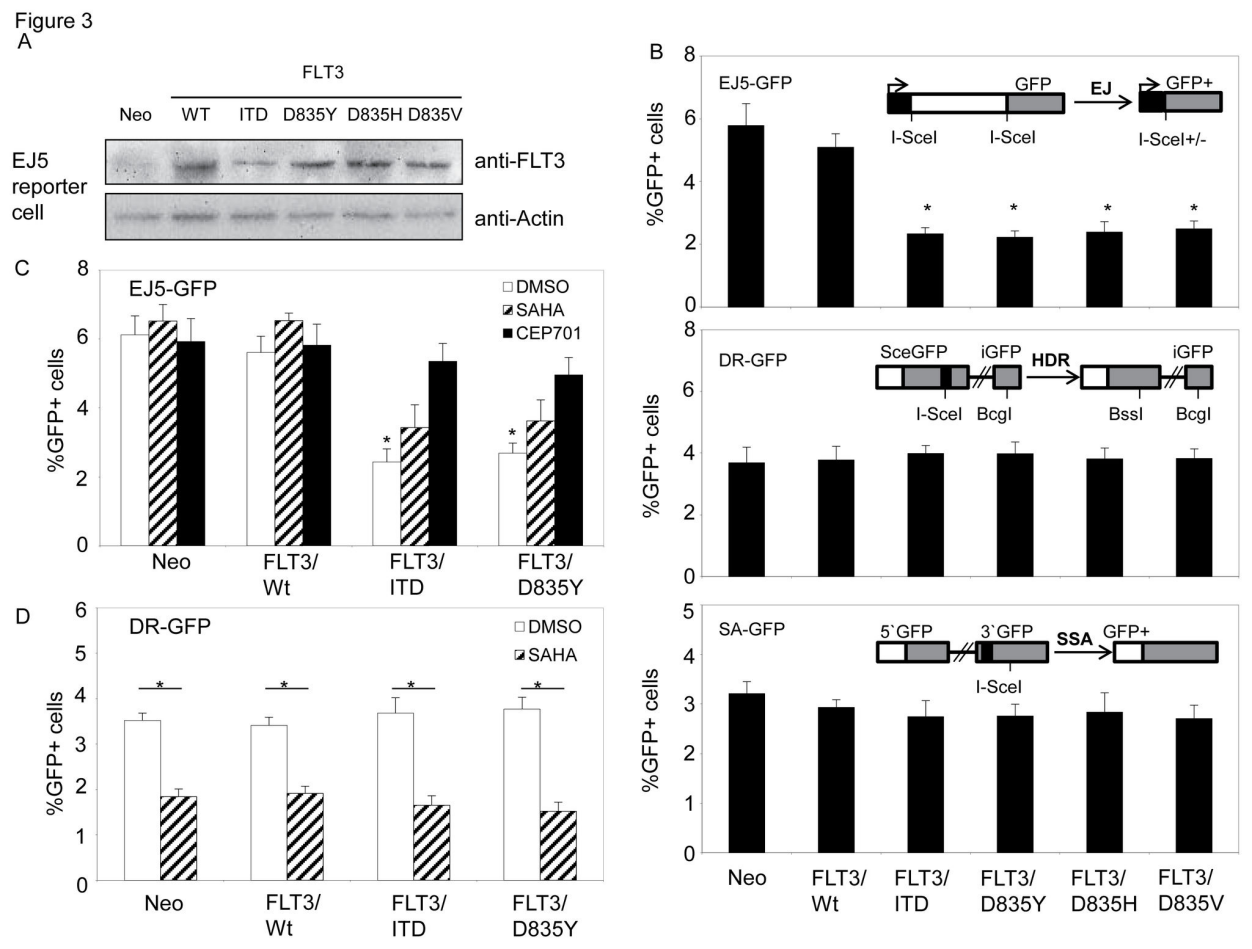
Expression of FLT3/ITD or FLT3/D835 mutation inhibits NHEJ activity in response to DSB DNA damage. (A) Transient expressions of wild-type or mutant FLT3 proteins in U2OS reporter cells. U2OS-EJ5 reporter cells were transiently transfected with plasmid constructs encoding wild-type or FLT3 mutants. Empty vector (neo) was included as transfection control. Whole cell extracts were prepared 24 hours later and analyzed by immunoblot assay with anti-FLT3 antibody. Anti-actin antibody was included for loading control. (B) Expression of mutant FLT3 inhibits NHEJ activity in GFP reporter cells. Plasmid DNA constructs with different forms of mutant FLT3 were co-transfected with I-SceI into U2OS GFP reporter cells. Repair of I-SceI-induced DSB is quantified by flow cytometry. Up to 5x10^4^ cells were counted for each sample. (C) Effects of SAHA and CEP701 on mutant FLT3-induced inhibition of NHEJ. U2OS/EJ5 cells were co-transfected with I-SceI and FLT3 constructs, and exposed to either 200 nM SAHA or 200 nM CEP701. Repair of I-SceI-induced DSB is quantified by flow cytometry. DMSO was included as control. (D) Effects of FLT3 mutants on SAHA-reduced HDR. U2OS/DR cells were co-transfected with I-SceI and FLT3 constructs, and exposed to 200 nM SAHA. Repair of I-SceI-induced DSB is quantified by flow cytometry. DMSO was included as control. Data represents the average of three experiments. Error bars indicate standard deviation. * indicates significance (P<0.05).

To address whether expression of mutant, constitutively active FLT3 would affect radiation response, we utilized a set of GFP-based reporter assays in sarcoma U2OS cells that allow measurements of distinct DSB repair pathways [15]. We transiently expressed different forms of FLT3 together with I-SceI in these reporter cells, and measured GFP+ cells after 72 hours. Overexpression of wild-type FLT3 did not cause a statistically significant change in repair frequencies for end-joining (EJ5-GFP), HDR (DR-GFP), or SSA (SA-GFP). In contrast, transient expression of either FLT3/ITD or FLT3/D835 mutants significantly reduced the frequency of end-joining repair, but did not cause a statistically significant change in HDR or SSA. Exposure to CEP701, a specific FLT3 kinase inhibitor, led to recovery of end-joining repair frequency in cells with expression of the FLT3 mutants (FLT3/ITD and FLT3/D835). We also found that treating cells with SAHA caused a decrease in the frequency in HDR regardless of expression of wild-type FLT3 or mutant versions, but did not affect mutant FLT3-compromised end-joining frequencies (Figure 3). 

**Figure 4:**
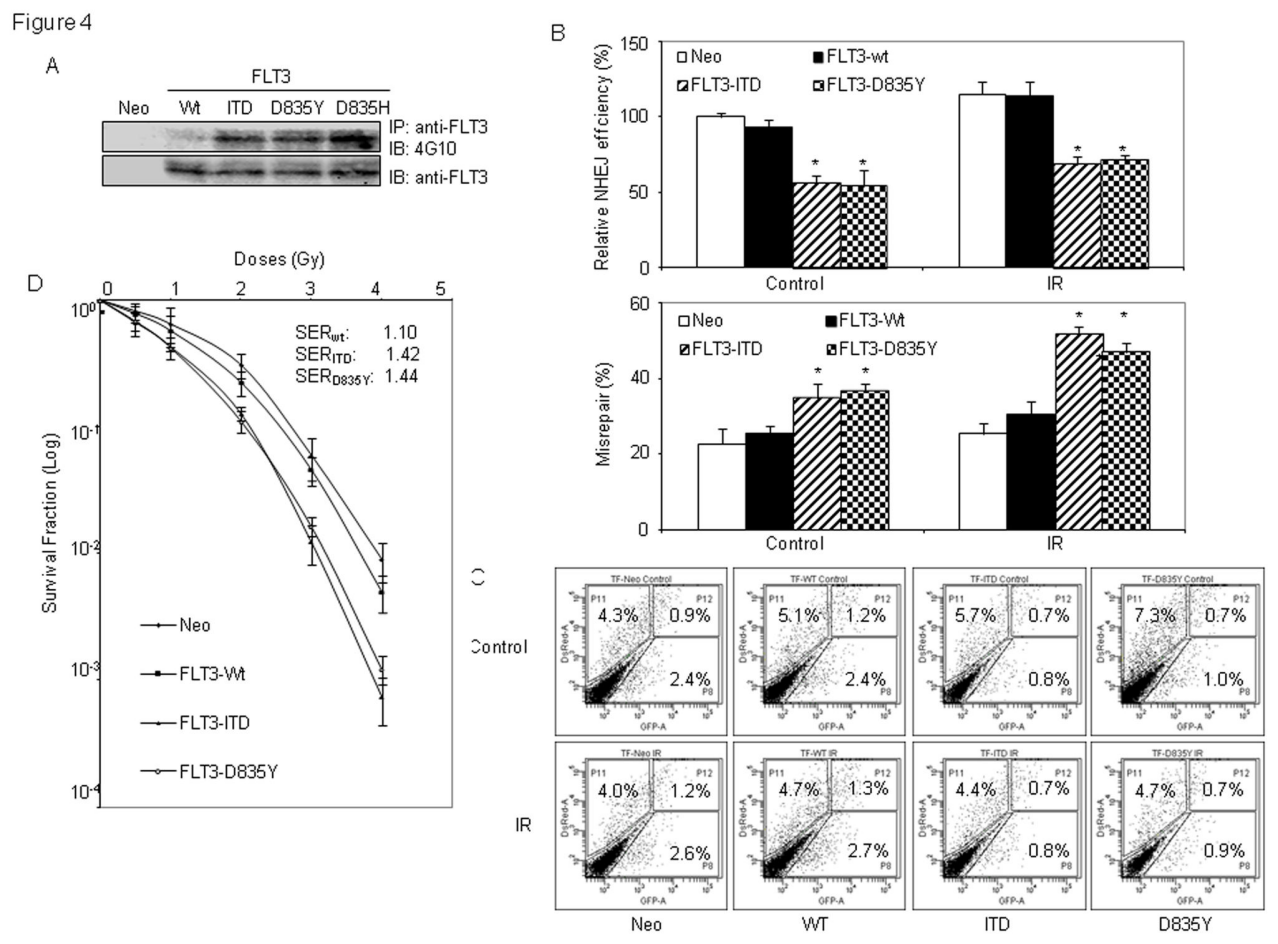
Expression of FLT3/ITD or FLT3/D835 mutation decreases the efficiency and fidelity of end-joining repair, and cause hypersensitivity to IR in AML cells. TF1 cells were engineered with stable expression of wild-type FLT3, or different forms of mutant FLT3, or empty vector as control. (A) Mutant FLT3 proteins with ITD or FLT3/D835 mutation are constitutively activated. Whole cell extracts were prepared and immunoprecipitated (IP) with anti-FLT3 antibody. Immunoblot assay (IB) was performed with anti-phosphotyrosine 4G10 antibody (upper panel). The membrane was then stripped and re-probed with anti-FLT3 antibody (lower panel). (B and C) Engineering expression of mutant FLT3 protein reduces the *in* *vivo* end-joining efficiency and increases misrepair frequency in TF1 cells. (B) Engineered TF1 cells were co-transfected with linear pUC18 and circle pDsRed as control. Cells were either irradiated with 1.2 Gy or untreated. Plasmid DNAs were then recovered from cells 72 hours later, and transformed into E.Coli for white/blue clone screening. End-joining efficiency (top) and misrepair frequency (bottom) were calculated according to the numbers of colony formed as described in Methods and Materials. Diagram shows changes of the *in* *vivo* end-joining efficiency and misrepair frequency; (C) as an alternative approach, engineered TF1 cells were co-transfected with linear EJ5-GFP reporter and circle pDsRed and irradiated. GFP+ cells were measured by flow cytometry assay, and the ratios of GFP+ / RED+ cells were calculated to determine the end-joining repair efficiency. Flow cytometry results represent one of three independent experiments. (D) AML cells expressing constitutively activated mutant FLT3 are more sensitive to IR. Engineered TF1 cells were treated with 200nM SAHA followed by IR. Clonogenic survival assay were performed as described in materials and methods. Data represents the average of three experiments. Error bars indicate standard deviation. * indicates significance (P<0.05).

We further examined the effects of these FLT3 mutants on NHEJ efficiency and the frequency of misrepair in AML cells. For this, we generated stable cell lines expressing wild-type or different forms of mutant FLT3 in FLT3-null TF1 cells (Figure S3), and transiently transfected cells with linear plasmid pUC18 or EJ5-GFP together with circle pDsReD-Express2-N1 as transfection control. NHEJ repair efficiency was determined either by colony formation assay or by flow cytometry assay with measuring the ratios for re-cyclization of corresponding linear plasmid DNA, and misrepair frequency was determined by white/blue colonies in colony formation assay. We found that mutant FLT3 proteins were constitutively tyrosine phosphorylated in engineered TF1 cells (Figure 4A). Compared to control cells with engineering of empty vector, expression of wild-type FLT3 did not cause statistically significant changes in the frequencies of NHEJ repair or misrepair in TF1 cells. However, expression of either FLT3/ITD or FLT3/D835 mutants resulted in approximate 50% reductions in end-joining repair frequency, and statistically increased repair errors by 1.37 fold for FLT3/ITD and 1.45 fold for FLT3/D835Y as well. When cells were treated with 1.2 Gy IR, reduced NHEJ efficiency and elevated misrepair frequency with statistically significances were also observed in cells expressing mutant FLT3 proteins when compared to either FLT3-null cells or cells with wild-type FLT3 (Figure 4B and C, and Figure S4). 

These results indicated that constitutively activated FLT3 with ITD or D835 mutations compromises NHEJ repair, which may cause the diminished survival in AML cells after radiation treatment. In supporting this, clonogenic survival assays showed that TF1 cells with engineering expressions of FLT3/ITD or FLT3/D835Y were more radiation sensitive than parental TF1 cells or cells with expression of wild-type FLT3 (Figure 4D). We also noticed that MV4-11 cells that expressing FLT3/ITD mutation were more radiosensitive than TF1 (FLT3-null), and THP1 or KG1A (wild-type FLT3) cells (Figure 1C) 

### Expressions of FLT3 mutants block DNA-PKCs activation in response to IR

**Figure 5:**
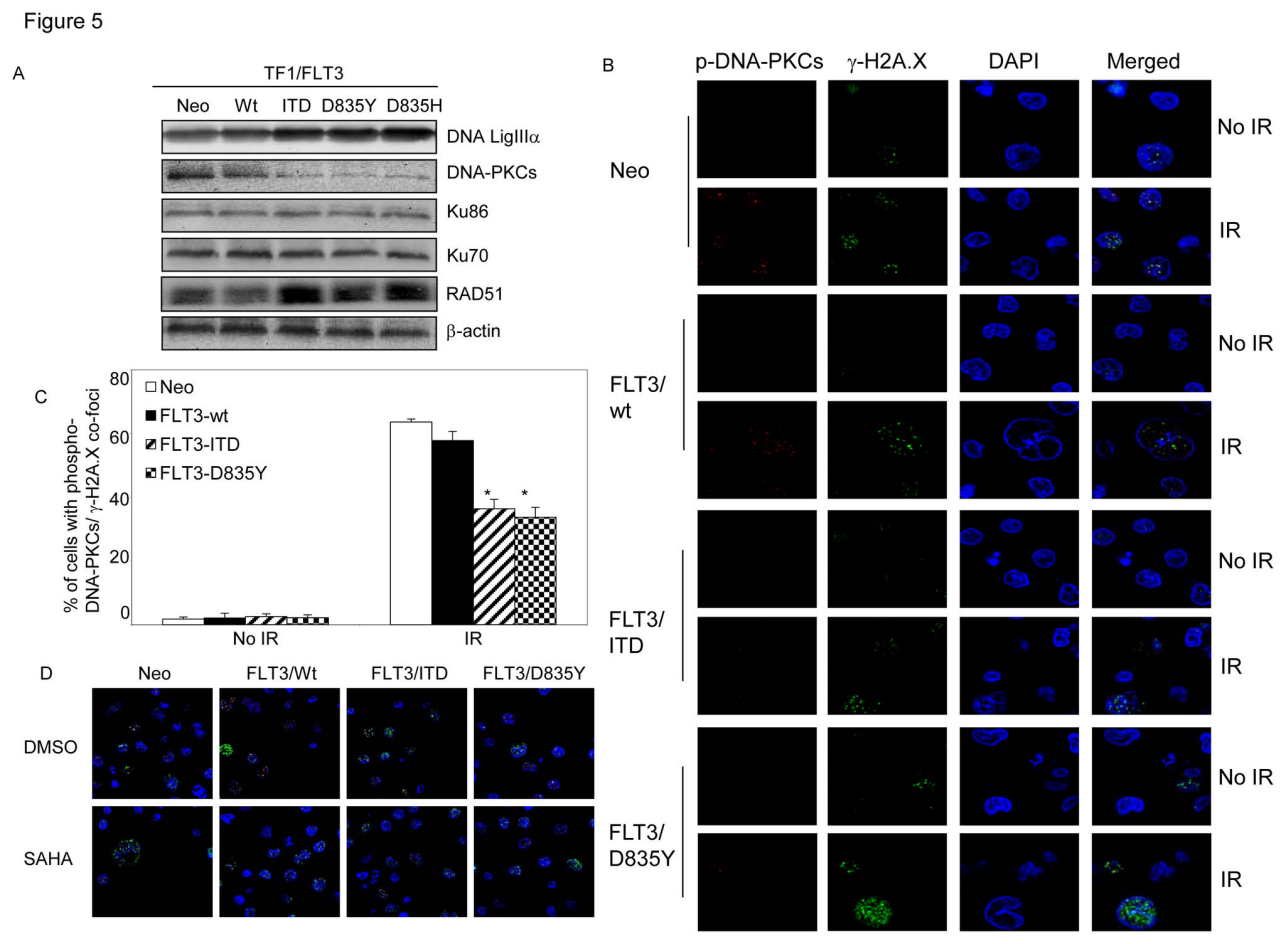
The effects of constitutively activated FLT3 mutants on end-joining DNA repair are associated with inhibited DNA-PKCs activity. (A) Immunoblot assay. Whole cell extracts from TF1 cells with stable expression of different forms of FLT3 were analyzed by immunoblot assays with anti-DNA LigIII, DNA-PKCs, Ku70, Ku86 and RAD51 antibodies. β-actin was included to confirm equivalent protein loading. (B) Representative images of the changes for nuclear phosphor-DNA-PKCs /γ-H2A.X co-foci in response to IR treatment. Engineered TF1 cells were irradiated with 1.2 Gy. Cells were collected one hour later for immunofluorescence staining with anti-γ-H2A.X (green) and anti-phosphor-DNA-pK (T2609, red). Nuclei were stained with DAPI (blue). Images were acquired with LSM 510 confocal microscope (Zeiss) with 80X objective and processed by Photoshop (Adobe). (C) Diagram shows changes in the fraction of cells with phosphor-DNA-PKCs/γ-H2A.X co-foci in engineered TF1 cells. (D) Expressing FLT3 mutants does not change the inhibitory effects of SAHA on the formation of RAD51/γ-H2A.X nuclear co-foci in irradiated TF1 cells. Engineered TF1 cells were collected 7 hours later after 1.2 Gy of irradiation, and immunofluorescence stained with anti-γ-H2A.X (green) and anti-RAD51 (red). Nuclei were stained with DAPI (blue). Error bars indicate standard deviation. * indicates significance (P<0.05).

Studies showed that expressing FLT3/ITD mutation altered several NHEJ pathway components in mouse pro-B lymphocyte BaF3 cells, including up-regulation of DNA Lig IIIα protein and down-regulation of Ku70/Ku86 proteins [16,29]. We also found that expressions of FLT3/ITD, FLT3/D835Y or FLT3/D835H in TF1 cells resulted in elevated DNA Lig IIIα expressions, which may contribute to the observed error-prone DNA repair as shown in Figure 4B [16,30]. However, no changes in protein levels of Ku70 or Ku86 were observed. Instead, we noticed significant decreases of DNA-PKCs in these cell populations (Figure 5A). 

To outline the potential role of DNA-PKCs in DNA damage repair in mutant FLT3-modulated radiation response, we analyzed the changes of the formation for phosphor-DNA-PKCs/γ-H2A.X nuclear co-localization in engineered TF1 cells in response to IR. As shown in Figure 5B and C, only 2-3% of cells showed baseline phosphor-DNA-PKCs/γ-H2A.X co-foci in all cell strains. IR treatment dramatically induced the formations of phosphor-DNA PKCs/γ-H2A.X co-foci in control cells: at one hour post-IR, the percentages of cells with co-foci were 63.6 ± 0.93 for FLT3-null control cells and 57.8 ± 1.85 for cells with wild-type FLT3, respectively. However, the percentage of co-foci positive cells was statistically lower in cells with either FLT3/ITD (36.4 ± 2.91, p=0.014 when compared to TF1 cells with wild-type FLT3) or FLT3/D835Y (33.7 ± 3.11, p=0.017), indicating expression of these FLT3 mutants blocked the activation and recruitment of DNA-PKCs to damaged DNA sites. 

### SAHA induces hypersensitivity to IR in AML cells expressing constitutively activated FLT3

It is now accepted that there is crosstalk between DNA damage repair pathways and that if one pathway is compromised, compensatory repair occurs via other pathways. Thus, if NHEJ efficiency is compromised in AML cells with FLT3 mutants, the HR pathway may be up-regulated. Indeed, study has shown that constitutive activation of FLT3/ITD led to up-regulation of RAD51, which may result in resistance to therapy in AML [31]. We also observed increased RAD51 protein level in engineered TF1 cells with expression of mutant FLT3/ITD or FLT3/D835 (Figure 5A). However, we noticed that expressing FLT3/ITD or FLT3/D835Y did not affect the SAHA-inhibited the nuclear formation of RAD51/γ-H2A.X co-foci in irradiated TF1 cells, indicating that SAHA can suppress HDR in response to IR in AML cells where NHEJ is already compromised by expression of these FLT3 mutants (Figure 5D). 

**Figure 6:**
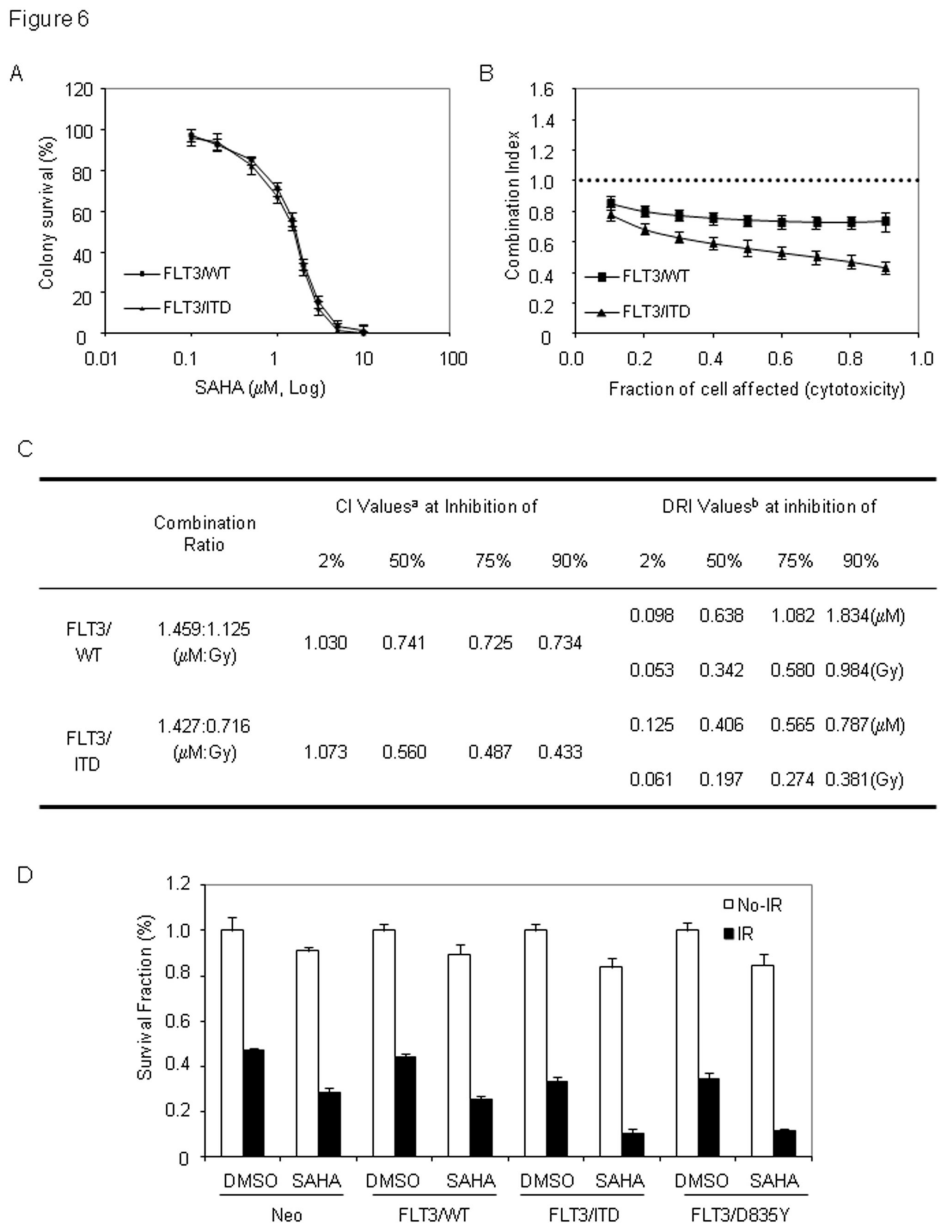
AML cells expressing constitutively activated FLT3 mutants are hypersensitive to the radiosensitizing effect of SAHA. (A) Engineered TF1 cells were treated with varying doses or SAHA and plated for colony formation assay to determine LD_50_. (B) Median Effect assay. Cells were exposed to combined treatment of SAHA and IR with constant ratios, and plated for colony formation assays as described in materials and methods. CIs were calculated from at least three separate experiments, and averages were plotted versus fraction of cells affected. (C) Median Effect Analysis. a: CI<1, CI=1, and CI>1 indicate synergism, additive effect, and antagonism, respectively; b: DRI represents the order of magnitude (fold) of dose reduction that is allowed in combination for a given degree of effect compared with the dose of each treatment alone. Upper values are for SAHA (μM) and lower values are for IR (Gy). (C) Expression of mutant FLT3 enhances the inhibitory effect of SAHA on clonogenic survival in irradiated TF1 cells. Engineered TF1 cells were pretreated with 500 nM SAHA for 16 hours, and then irradiated with 1.2Gy IR. Clonogenic survival assay were performed as described in materials and methods. Diagram shows changes in the survival fractions of cells. Error bars indicate standard deviation. * indicates significance (P<0.05).

We thus hypothesize that SAHA exposure may induce more radiosensitization in AML cells expressing mutant FLT3. For this, we employed median effect analysis, which allows potential interactions to be quantitatively studied over the complete range of cells affected by the combined treatment [18,32]. We first measured LD_50_ values for the individual agents by colony formation assay. Expressing FLT/ITD increased IR-induced toxicity and reduced LD_50_ value (to 0.716 Gy) in TF1 cells, when compared to cells expressing wild-type FLT3 (LD_50_=1.125 Gy, Figure 4B). However, a nearly identical LD_50_ (^~^1.45 μM) for SAHA were observed (Figure 6A). After exposure to combined treatment with constant ratios, synergistic radiosensitization effects of SAHA (with CI values of < 1) were clearly demonstrated for both cell populations. However, compared to cells expressing wild-type FLT3, where CI values remained almost constant between 0.86 to 0.72 over the entire range of cells affected, cells expressing FLT/ITD showed a CI curve with lower CI values, which also kept decreasing as the fraction of cells affected increased (Figure 6B and C). 

In addition, we noticed that the CI values for SAHA at lower concentration (^~^ 100nM) were above 1 in both cell lines. Interestingly, SAHA at 100nM does not induce obvious acetylation of histone H4 in TF1 cells (Figure 1B), which suggests histone acetylation may have an important role in SAHA-induced synergistic radiosensitization. 

We further tested the effects of these constitutively activated FLT3 mutants on SAHA-modulated radiation responses in TF1 cells exposed to a single TBI clinical radiation dose (1.2 Gy). In this experiment, we pretreated cells with 500 nM SAHA: at this dose SAHA might affect 50% cells when combined with IR (Figure 6C). As expected, SAHA exposure reduced survival rates in irradiated FLT3-null cells (from 46.95 ± 1.32 to 28.48 ± 2.14, p=0.027), and in cells with wild-type FLT3 (from 44.28 ± 1.43 to 25.47 ± 1.86, p=0.031). In cells expressing either FLT3/ITD or FLT3/D836Y, however, SAHA exposure led to more significant decreases of survival rates (from 33.10 ± 2.61 to 10.25 ± 2.12 for FLT3/ITD and from 34.77 ± 2.24 to 11.40 ± 1.32 for FLT3/D835Y, respectively). Despite the slight inhibitory effects of SAHA on colony formation, nearly 1.8-fold comparable reductions, with statistically significances, in colony survival in response to combined treatment were observed in cells with these FLT3 mutants when compared to FLT3-null cells or cells with wild-type FLT3 (Figure 6D).

Taken together, these results indicate synergistic radiosensitization effects of SAHA are further enhanced in cells with mutant, constitutively activated FLT3. 

## Discussion

AML is an aggressive malignancy affecting mostly older patients. Though AML is responsive to first-line chemotherapy, and adults presenting with newly diagnosed AML will have a 60–70% chance of attaining a remission, only 20–40% of patients will attain a lasting remission [33]. For patients with relapse, only HCT has been shown to be potentially curative [33,34]. Though a broad spectrum of regimens has been studied for optimization, regimen-related toxicities have restricted the use of HCT to young and relatively healthy patients, however [35,36].

TBI is a mainstay of the conditioning regimens for HCT for AML patients. The primary reasons for using TBI in HCT include tumor cell eradication and immunosuppression to allow for engraftment of allogeneic donor marrow. To this setting, development of radiosensitizer may help to kill AML cells, but not the latter, to improve therapeutic efficacy for AML patients. In addition, TBI provides therapy to sanctuary sites not easily reached by chemotherapy drugs and provides another mechanism of tumor cell kill against chemotherapy-resistant cell clones [37]. When administered in doses of 1000 to 1500 cGy, TBI is clearly effective in the treatment of AML. Randomized phase II trials have demonstrated the relapse following transplantation was significantly reduced with the higher dose of TBI; however, the non-relapse mortality was increased with the higher dose of TBI, and consequently, survival in the two arms was equivalent [38]. Therefore efforts to deliver a more targeted form of radiotherapy as part of the conditioning regimen, using radiolabeled antibodies or using intensity modulated radiotherapy to selectively reduce doses to critical organs and increase doses to target structures such as bone marrow are important to allow for the potential to dose escalate with acceptable toxicities. In the setting of targeted radiotherapy, radiosensitizing agents become clinically important in potentially further amplifying radiotherapy effects of leukemia cells over effects of normal tissues.

Histone deacetylases (HDAC) remove acetyl groups from core histones and nonhistone proteins, and play an important role in many diverse and essential biological processes [39]. Inhibitors of HDAC, HDIs, are promising drugs that lead to growth inhibition, cell cycle arrest, premature senescence, apoptosis and radiosensitization in a broad spectrum of human malignant cells [40]. As an orally administered HDI with a manageable profile of side-effects and preliminary evidence of anti-tumor activity, SAHA (vorinostat) was the first HDI approved by the US Food and Drug Administration for treating cutaneous T-cell lymphoma (CTCL) and is being tested for other malignancies [41]. SAHA, both as a single agent and in combination with other agents, has shown antitumor activity in preclinical models and proven its efficacy in patients with AML [42,43]. Recent studies have indicated that SAHA induced reactive oxygen species and DNA damage in AML cells, suggesting a potential role of SAHA in combination therapies with IR and/or other DNA damaging agents for AML patients [28,44]. Very recently, a preclinical study also showed that SAHA modulated proliferation and self-renewal properties of leukemic stem and progenitor cells (LSC), which may cause LSC to be more sensitive to chemotherapy [45]. However, the characterization of SAHA in therapeutic strategy for AML patients still remains incomplete. In this study, we demonstrated that SAHA at low doses could sensitize AML cells in vitro to IR through blockade of RAD51-dependent HDR. 

Previous study reported that HDACi could induce acetylation of Ku70, leading to mitochondria-dependent apoptosis; Study also showed that SAHA at high concentration (5µM) down-regulated protein expressions of Mre11 and RAD50, and induced DNA damage in transformed cells [46,47]. In our study, we also found that SAHA at high concentration (µM) could decrease protein expressions of RAD50, Mre11, KU70 and RAD51, indicating that SAHA may affect DSBs repair through multiple mechanisms. However, our data showed that SAHA at low concentration with minimal cytotoxicity (200 nM) had no effect on transcriptional regulation of RAD51 gene, yet it could posttranslationally modify RAD51 response to IR treatment and reduce HDR in irradiated AML cells. Furthermore, our data clearly showed that AML cells expressing constitutively activated FLT3 mutants were hypersensitive to the treatment of IR in the presence of low concentration of SAHA. 

FLT3 is normally expressed by hematopoietic stem/progenitor cells and as hematopoietic cells differentiate FLT3 expression is lost [48]. FLT3 is overexpressed in most AML patients, and constitutes a potentially attractive therapeutic target in AML. It is also the most commonly mutated gene in AML accounting for 30-35% of de novo cases [49,50]. Mutations in FLT3 receptor, in the form of ITD mutation in the juxtamembrane domain or single-base point mutations within the receptor tyrosine kinase domain, induce constitutive tyrosine kinase activity in the absence of the natural FLT3 ligand and confer growth factor independence, increased proliferation, and survival to myeloid precursor cells [25,49,51]. Patients with these mutations have a very poor prognosis, higher risk of relapse and lower leukemia-free survival [25,52,53]. . Targeting FLT3 tyrosine kinase with small molecule inhibitors has shown activity in preclinical models and in clinical trials. However, the majority of patients display primary or secondary resistance to FLT3 inhibitors [54-56]. In addition, acquisition of constitutively activated FLT3 mutations may result in an increase in DSBs and inaccurate repair by the error-prone NHEJ pathway, which in turn leads to genomic instability that has the potential to generate further genomic changes and facilitate leukemic disease resistance. For this reason, efficiently eliminating these aggressive AML cells will have clinic importance for disease control. Thus, our data present here suggest a potential clinic impact for use of SAHA as a part of radiotherapy containing conditioning regimen for helping radiotherapy to kill AML cells, and patients with mutant FLT3 receptor may be further benefited from this therapeutic strategy. Future plans are to evaluate the effects of SAHA on radioresponses in primary AML samples to verify the findings on cell lines. In addition, whether SAHA at certain low concentrations may protect AML cells, or AML cells with mutant FLT3, from IR needs to be further studied.

## Supporting Information

Figure S1 **Dose-dependent cytotoxicity of SAHA on AML cells *invitro*.** AML cells were treated with indicated concentrations of SAHA, and plated for colony formation assay to determine the cytotoxicity of SAHA.
(TIF)

Figure S2 A-C. SAHA induces persistence of γ-H2AX, and inhibits the formation of RAD51/γ-H2AX co-foci in irradiated AML cells. A and B. Representative images of nuclear RAD51 /γ-H2A.X co-foci in irradiated THP1 cells. THP1 cells were exposed to vehicle (DMSO, A) or 200 nM SAHA (B) for 16 hours and irradiated (1.2 Gy). Cells were then collected for immunofluorescence staining with anti-γ-H2A.X foci (green) and anti-RAD51 (Red). Nuclei were stained with DAPI (blue). Images were acquired with LSM 510 confocal microscope (Zeiss) with 40X objective and processed by Photoshop (Adobe). C. Representative images of nuclear RAD51 /γ-H2A.X co-foci in irradiated MV4-11 cells. MV4-11 cells were exposed to 200 nM SAHA for 16 hours and irradiated (1.2 Gy). Cells were then collected for immunofluorescence staining with anti-γ-H2A.X foci and anti-RAD51. D. Diagram shows change in the fraction of cells with γ-H2A.X foci (left) and RAD51/γ-H2A.X co-foci (right) in three AML cell lines. E. Effects of SAHA on protein expressions. THP1 cells were exposed to indicated concentrations of SAHA for 16 hours (left), or 200 nM SAHA for upto 48 hours. Immunoblot assays were then performed to determine the effects of SAHA on the protein levels of DSBs repair-related proteins.
(TIF)

Figure S3 **Expression and activation of FLT3 in AML cells.** (A) RT-PCR; (B) immunoprecipitation assay.
(TIF)

Figure S4 ***invivo* end-joining assay.** Engineered TF1 cells were co-transfeced with linear EJ5-GFP and circle pDsRed as control, cells were irradiated with 1.2 Gy and analyzed for GFP+ cells by flow cytometry assay. End-joining efficiency was calculated according to the ratios of GFP+ / RED+ cells. Data represents the average of three experiments. Error bars indicate standard deviation. * indicates significance (P<0.05).
(TIF)
