# Supplementary figures and images for: Suberoylanilide Hydroxamic Acid Induces Hypersensitivity to Radiation Therapy in Acute Myelogenous Leukemia Cells Expressing Constitutively Active FLT3 Mutants
Source: PLoS One. 2013 Dec 19;8(12):e84515. doi: 10.1371/journal.pone.0084515 (PMC3868602; doi:10.1371/journal.pone.0084515)

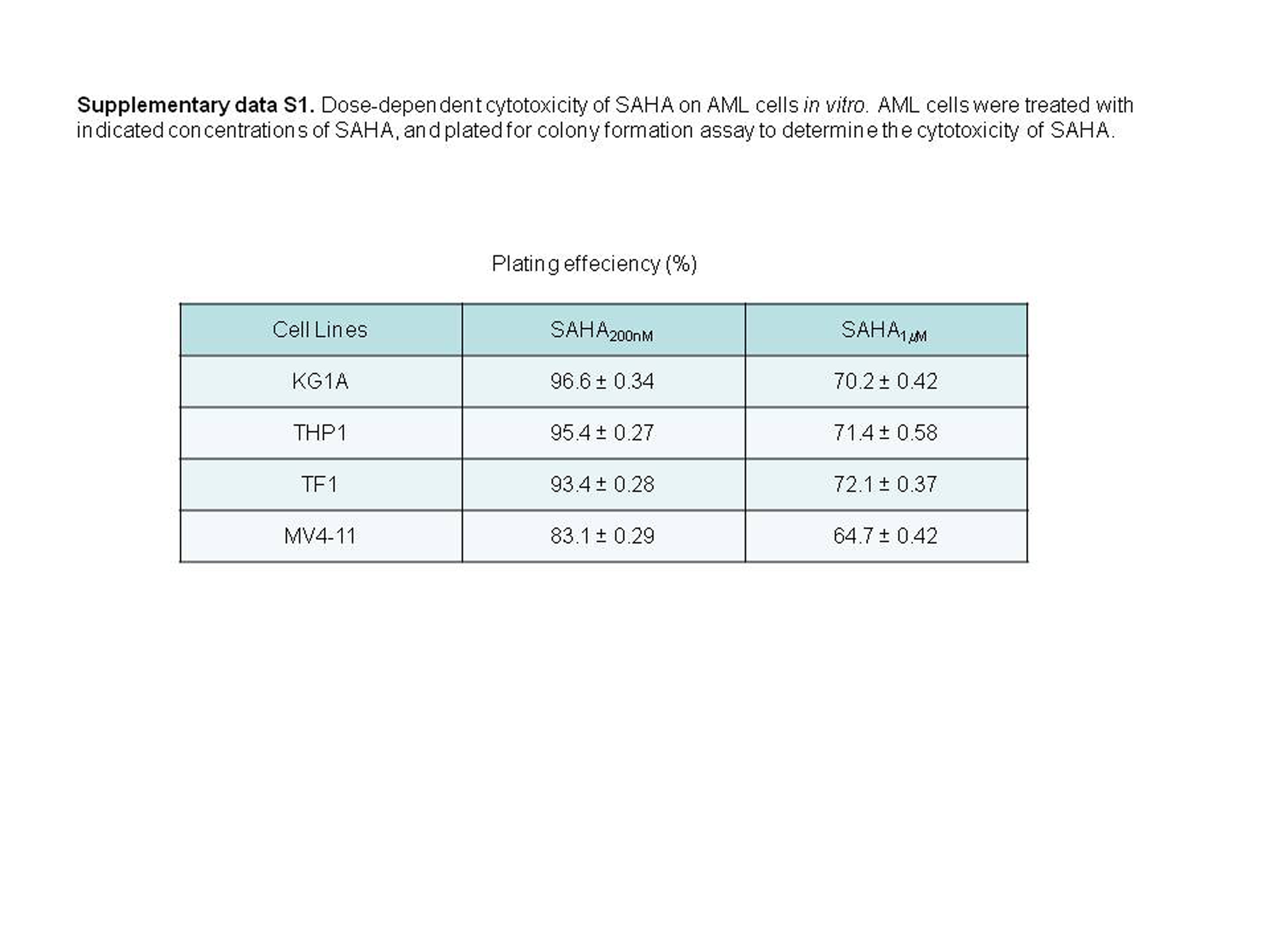

Supplement: Figure S1 — Dose-dependent cytotoxicity of SAHA on AML cells invitro. AML cells were treated with indicated concentrations of SAHA, and plated for colony formation assay to determine the cytotoxicity of SAHA. (TIF) [file pone.0084515.s001.tif]

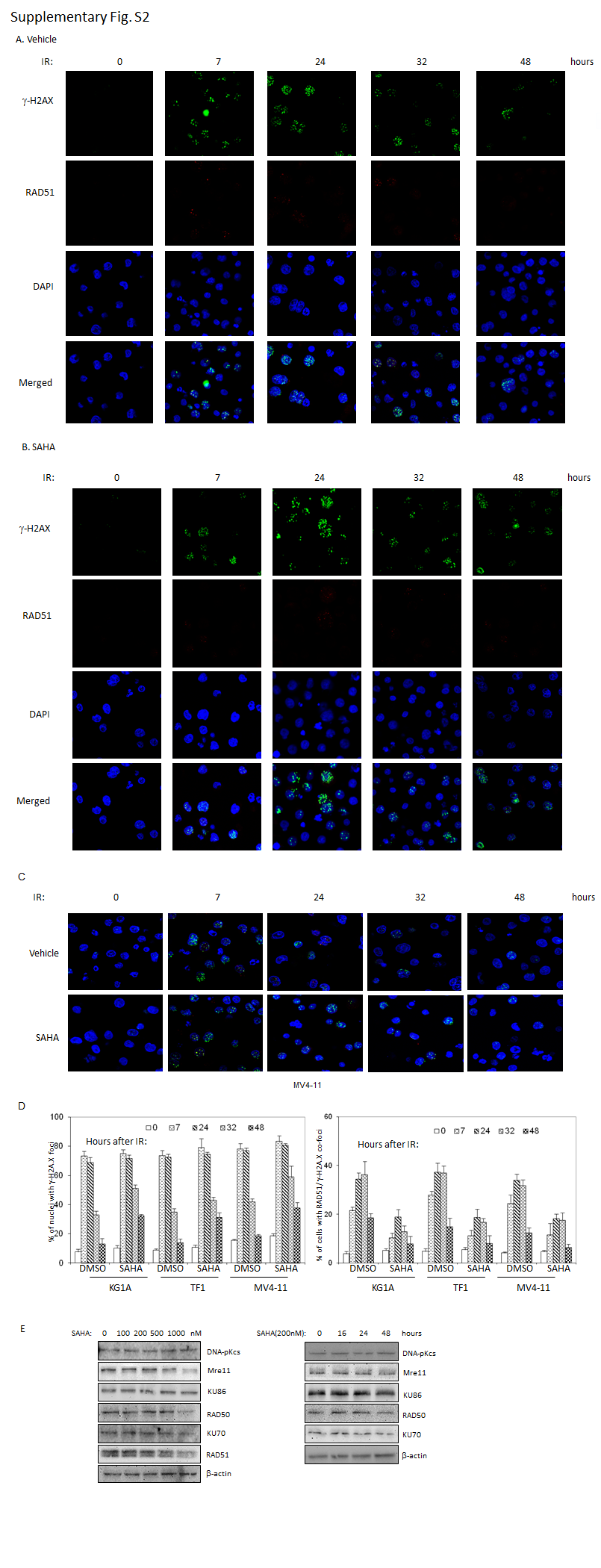

Supplement: Figure S2 — A-C. SAHA induces persistence of γ-H2AX, and inhibits the formation of RAD51/γ-H2AX co-foci in irradiated AML cells. A and B. Representative images of nuclear RAD51 /γ-H2A.X co-foci in irradiated THP1 cells. THP1 cells were exposed to vehicle (DMSO, A) or 200 nM SAHA (B) for 16 hours and irradiated (1.2 Gy). Cells were then collected for immunofluorescence staining with anti-γ-H2A.X foci (green) and anti-RAD51 (Red). Nuclei were stained with DAPI (blue). Images were acquired with LSM 510 confocal microscope (Zeiss) with 40X objective and processed by Photoshop (Adobe). C. Representative images of nuclear RAD51 /γ-H2A.X co-foci in irradiated MV4-11 cells. MV4-11 cells were exposed to 200 nM SAHA for 16 hours and irradiated (1.2 Gy). Cells were then collected for immunofluorescence staining with anti-γ-H2A.X foci and anti-RAD51. D. Diagram shows change in the fraction of cells with γ-H2A.X foci (left) and RAD51/γ-H2A.X co-foci (right) in three AML cell lines. E. Effects of SAHA on protein expressions. THP1 cells were exposed to indicated concentrations of SAHA for 16 hours (left), or 200 nM SAHA for upto 48 hours. Immunoblot assays were then performed to determine the effects of SAHA on the protein levels of DSBs repair-related proteins. (TIF) [file pone.0084515.s002.tif]

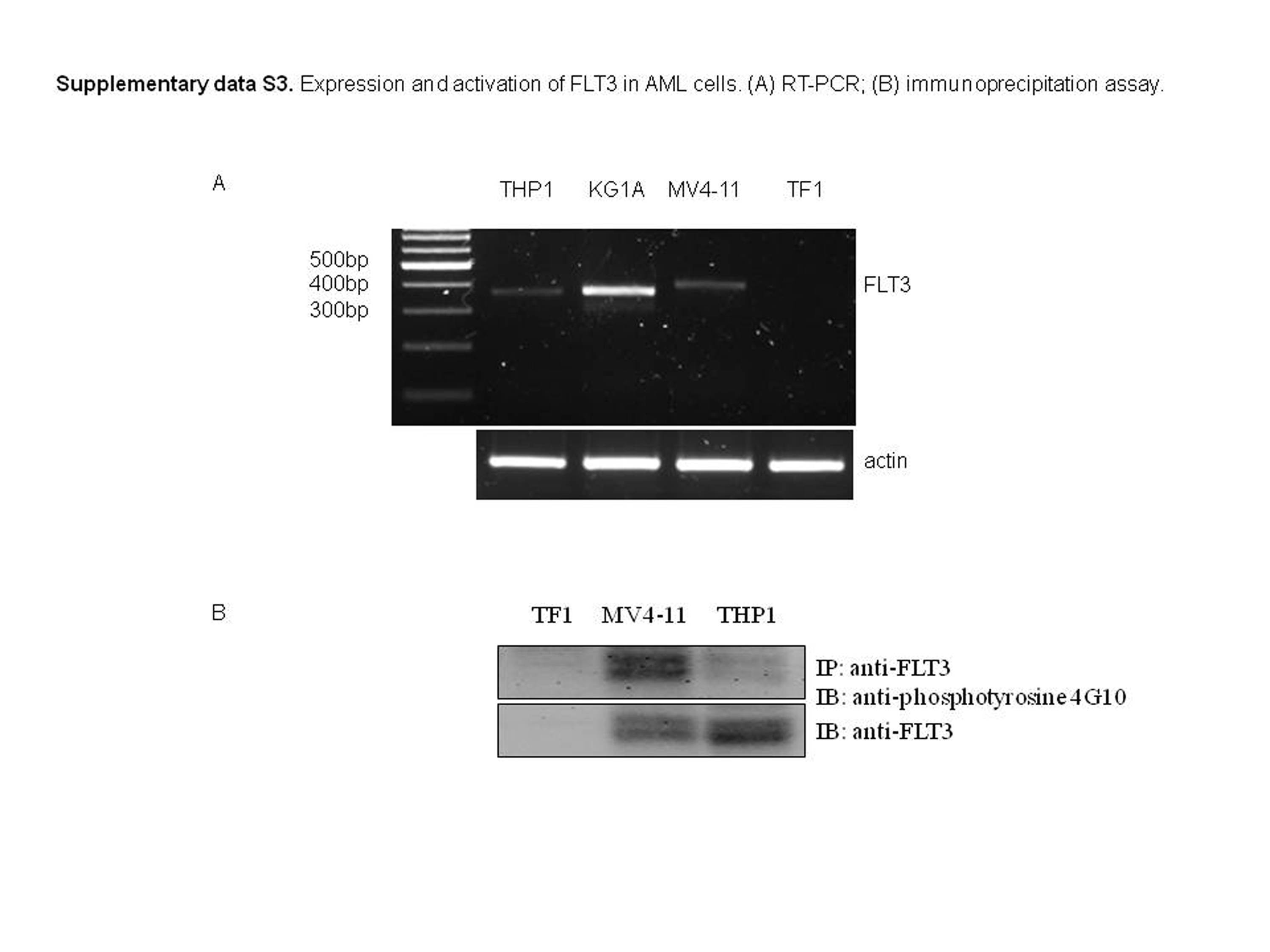

Supplement: Figure S3 — Expression and activation of FLT3 in AML cells. (A) RT-PCR; (B) immunoprecipitation assay. (TIF) [file pone.0084515.s003.tif]

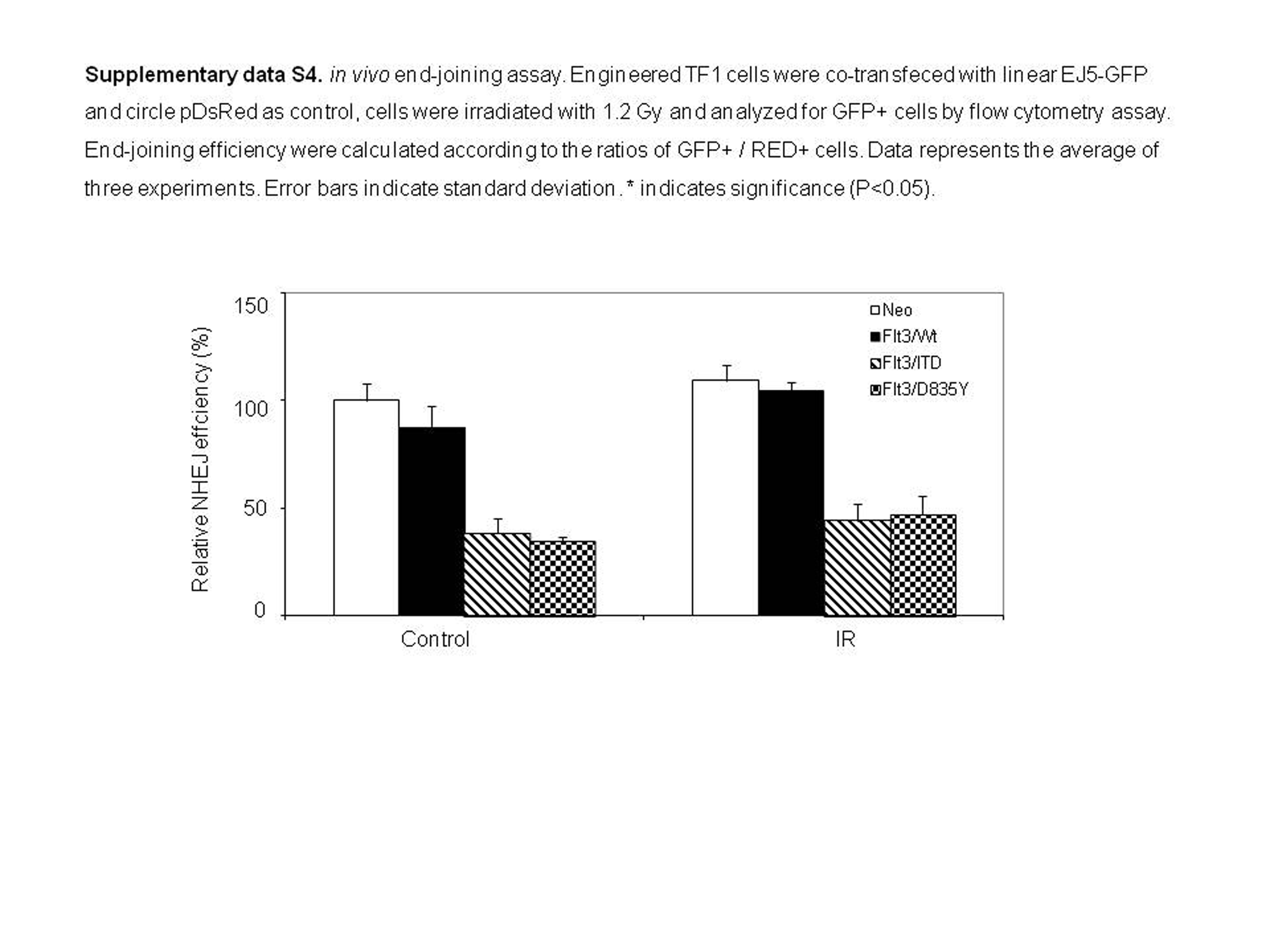

Supplement: Figure S4 — invivo end-joining assay. Engineered TF1 cells were co-transfeced with linear EJ5-GFP and circle pDsRed as control, cells were irradiated with 1.2 Gy and analyzed for GFP+ cells by flow cytometry assay. End-joining efficiency was calculated according to the ratios of GFP+ / RED+ cells. Data represents the average of three experiments. Error bars indicate standard deviation. * indicates significance (P<0.05). (TIF) [file pone.0084515.s004.tif]
